# Analyzing gender gap in agricultural productivity: Evidence from Ethiopia

**DOI:** 10.1016/j.jafr.2023.100960

**Published:** 2024-03

**Authors:** Takele Abdisa, Abule Mehare, Mekonnen B. Wakeyo

**Affiliations:** a Department of Economics, Addis Ababa University, Addis Ababa, P.O. Box: 1176, Ethiopia; b Policy Studies Institute/PSI, Addis Ababa, P.O. Box: 2479, Ethiopia; c Ethiopian Economic Association/EEA, Addis Ababa, P.O. Box 34282, Ethiopia; d Agriculture and Rural Development Study Center, Policy Studies Institute/ PSI, P.O. Box 2479, Ethiopia

**Keywords:** Gender, Productivity gap, Impact, Decomposition

## Abstract

Women make up more than 50% of the agricultural labor force but contribute less than 30% to agricultural productivity. Agriculture is the main source of livelihood in Ethiopia but by contributes more than 35% to GDP, 90% to forex earnings, and 70% to employment sources. So Improving economic well-being ensuring sustainable development, and reducing poverty is impossible by ignoring the role of women. Consequently, the main objective of the study is to investigate the gender gap in agricultural productivity using national Panel data of 3474 households which were collected in 2017 and 2019 by the Policy Studies Institute for AGP II baseline study and midline evaluation. Among these, 69% (2404) were male-headed and 31% (1070) were female-headed households. The panel data were combined with DID, Oaxaca Decomposition, and the Random effect Tobit model to investigate the gender gap. The result from the DID Method of Impact Evaluation shows that female-headed households were less productive by 3.7% and 2.05 quintals per hectare when measured in terms of value in birr and quintals per hectare compared to male-headed households. In contrast, male-headed households were more productive by 4% and 2.05 quintals compared to female-headed households. Additionally, the results from the pooled and random effect Tobit model showed that soil fertility, sex of the household head, slope of the land, total livestock holding in TLU, extension contact, use of inorganic fertilizer, credit use, machinery use, and plantation method are among the determinants of the gender gap in agricultural productivity. Furthermore, results from the Oaxaca decomposition show that a gender productivity difference between male and female-headed households was roughly 11.2% when measured by value and 5% when measured by an area-weighted formula. The main finding of the study is that endowment effects were less likely to have a significant impact on the productivity gap than structural effects did. Differences in the unexplained characteristics of men and women may also contribute to the considerable productivity gap between male-headed and female-headed households. Therefore, working on women's empowerment to improve their structural disadvantages through various training programs that favor women or gender-mainstreamed extension training programs for lowering gender productivity differentials is a possible policy option.

## Highlights

• Women make up more than 50% of the agricultural labor force in Sub-Saharan Africa, but they have less access to productive resources.
• Gender gap in agricultural productivity persist even after control for access to resource Endowment.
• Female headed household was, on average, 11.1% less productive than households with male headed.
• Endowment effects were less likely to have a significant impact on the productivity gap than structural effects did.

## Graphical abstract

**Figure:**
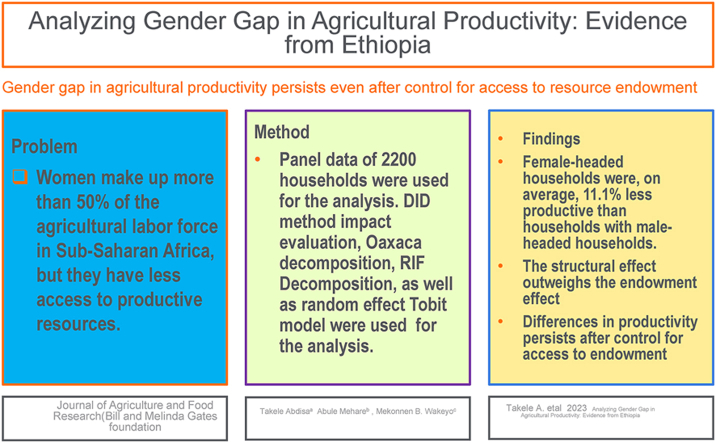

## 1 Introduction

In many Sub-Saharan African countries where smallholder farming predominates, agricultural output is often low and when comparing female farmers to their male counterparts, this difference is much more pronounced. Empirical evidence has consistently shown that one significant obstacle to the growth of the agricultural sector is the gender difference in agricultural production [1]. In Sub-Saharan Africa, women comprise over 50% of the agricultural labor force, with their contributions ranging from 4% to 40%, with the majority falling between 20% and 30% [[2], [3], [4]]. When compared to their male counterparts, female farmers' agricultural productivity is significantly lower in many Sub-Saharan African nations where smallholder farming is the majority [5,6]. Even after controlling for input levels and household characteristics, it is typically discovered that female farmers are less productive than male farmers [6]. Gender equality remains a crucial development objective for Ethiopia and the rest of the world as it is part of the 2030 Agenda for Sustainable Development's aim of empowering women and girls and achieving gender equality, but Ethiopia has poor performance on global gender indices [7]. The low agricultural output in SSA, including Ethiopia, is partly because women, who play a significant role, have little control over productive inputs like land, water, and cutting-edge technologies [8]. The gender disparities in agricultural production continue to be a significant barrier to the growth of the agricultural sector in Ethiopia [7]. Empirical evidence shows that women lack empowerment, in terms of having access to key factors of production, decisions about how to use the money they make, taking on leadership roles in their communities, and managing their time at home [9]. Gender-based inequities in the ownership and management of financially and economically productive resources impair agricultural production and undermine efforts to increase agricultural resilience and sustainability [10,11]. On the other hand, deeply rooted sociocultural norms and traditional gender roles frequently lead to unequal distribution [7]. Furthermore, Quisumbing & Doss [12] found that the time women spend working in agriculture may be impacted by other obligations they have in the home and community. The gender gap in agricultural productivity between male and female-headed households has been studied in the literature using different approaches. Among the methodologies used in the literature, Oaxaca Blinder is the most common method of decomposing the source of the Gender Gap. Decomposing productivity differentials to distinguish between resource ownership and returns to resources various approaches are employed [12].

The seminal work of Quisumbing [13] shed light on the gender gap in agricultural productivity and later [1,2,4,5,7,8,10,11,[14], [15], [16], [17]] have contributed to the area by estimating the share of the endowment effect and the structural impact to explain the gender gap in productivity using the Oaxaca-Blinder decomposition technique [18,19]. However, the Oaxaca Blinder decomposition method has some drawbacks, including the fact that it is susceptible to specification errors, lacks a counterfactual, and overestimates the importance of the endowment effect. It may also depend on the reference group used. The varying distribution of outcomes among the members of each group is not taken into account by the decomposition. It only offers details on the mean projected outcome difference between the two groups, which differs from the crude difference to the degree to which the two groups' distributions of other covariates change. The estimates of decomposition also differ according to which reference group is used. Usually, there is no compelling reason to select the best group. Additionally, reliable estimates via OB decomposition are only possible if the conditional expectation is linear and each covariate's contribution is dependent on the base selection of groups[20].

In light of this issue, estimating the conditional expectation via non-parametric techniques and utilizing a non-parametric reweighting method to enhance the decomposition process are potential solutions [21]. The non-linear decomposition method developed by Fairlie [22] is an extension of the linear decomposition techniques developed by Oaxaca [18] and Blinder [19] and is frequently used in investigations of the gender wage gap [23]. When the outcome variable is continuous, linear decomposition methods can be used to assess and explain result differences, including gender productivity differences. However, using linear decomposition techniques when outcome variables are not continuous may produce inaccurate and misleading results [16]. The main advantage of using the RIF-regression method in an Oaxaca-Blinder-type decomposition is that it provides a linear approximation of a highly non-linear function [21]. RIF regressions in combination with a reweighted strategy as a feasible methodology for decomposing differences in distributional statistics beyond the mean. This methodology has three advantages compared to other strategies in the literature: the simplicity of its implementation, the possibility of obtaining detailed contributions and individual covariates on the aggregate decomposition, and the possibility of expanding the analysis to any statistic for which a RIF can be defined [24].

The issue of the gender gap in agricultural productivity attracted the interest of many scholars. For instance, Aguilar et al. [1] in Ethiopia, Kilic et al. [8], in Malawi, Makate and Mutenje [16] in Tanzania showed 13.4%, 82%, 70.3%, and 94% of this difference in productivity gap is explained by observable characteristics, or resource endowment respectively. Many of these researches suggest that estimates of the gender productivity gap become small after disparities in access to productive resources and personal traits are taken into account. Others revealed that the gap is driven majorly by differences in returns to resource endowment/structural effect between male- and female-headed households. For instance, Hirpa Tufa et al. [10] in Malawi, Ali et al. [4] in Uganda, and Bello et al. [11] in Nigeria showed 23.1%, 30.4%, and 77.6% of the difference in productivity gap is explained by unobservable characteristics or returns to resource endowment respectively.

Apart from the decomposition strategy, other researchers, such as Regasa et al. [25] used a two-stage least squares regression model, which is not effective in identifying the underlying causes of gender disparity. Furthermore, Quisumbing [26] used the OLS model, Bonis-Profumo et al. [27] used the log-log model, and Peterman et al. [6] used the multivariate Tobit model to study the gender gap in agricultural productivity. These studies, however, were unable to identify the underlying factors influencing the gender gap in agricultural productivity because they were more heavily reliant on linearly restricted assumptions. However, other authors argue that the productivity gap would persist even if women share the same characteristics as men, have equal access to resources for generating income, and are taken into account when making policy decisions [8,11,28]. The gender gap shrinking inside the family may provide women more power, enhancing their access to productive inputs, increasing their responsibility for decision-making and bargaining power, and enhancing their ability to make their own decisions that are in their economic interest [3,29]. It has been shown that the gender difference in agricultural productivity is related to either the gender of the household head or the gender of the person who manages the farm at home utilizing data and outcomes at the household level [10]. There are two components to the variation in productivity that may be separated: an endowment component (induced by inputs and other household variables) and a second component (caused by structural variations and unaccounted for the part) [30]. Therefore, to address the root causes of gender differences in productivity results, initiatives to eliminate the gender productivity gap should go beyond seeking to provide equal access to the resources that are already available [10].

The multiplier effect of increasing women's incomes through improved access to agricultural technologies is greater than that of increasing men's incomes, and it is crucial to raise food and nutrition security for families to raise living standards for future generations [12]. Increasing the economic viability of women farmers leads to better infant and child health indicators when women control additional income, as they tend to allocate more of their earnings toward the health and well-being of their children. This means that closing the agricultural gap is a proven strategy for enhancing the food security, nutrition, education, and health outcomes of children. Better-fed, healthier children learn better and become more productive citizens, and the benefits would span generations and pay large dividends in the future. Reversing the existing situation is a must for policymakers by implementing a combination of economic and behavioral reforms to achieve sustainable development goals, and it is hard to achieve sustainable development goals without increasing the productivity of women, who make up 50% of the population [30]. In doing so, gender analysis in agriculture sheds light on how socially constructed roles and duties influence a wide range of choices, from agricultural production and processing to market involvement to consumer choice and well-being results [12].

Even though the above-mentioned studies contribute to the body of literature, none of the studies evaluated the gender gap using panel data collected from the same household at various times to account for time-invariant characteristics. In addition, empirical research on gender productivity in Ethiopia is extremely limited, and it receives little academic support. Consequently, the general objective of the study was to analyze the gender productivity gap and identify the determining factors of the gap in Ethiopia, specifically to examine the gender discrepancy in agricultural productivity and identify the sources of the gender differential in agricultural productivity. This research work would be unique in using panel data and applying a combination of different econometrics models from previous studies, which aims to fill an empirical gap on the magnitude and sources of the gender gap in Ethiopia by employing nationally representative data that were collected from regions of high resource potential. As a result, this study examined factors that account for the gender productivity gap using the Difference in Difference (DID) method of impact evaluation^1^ and the random effect Tobit model to capture the impact of unobserved heterogeneity between male-headed and female-headed households. Additionally, the researchers used Oaxaca and RIF Oaxaca decomposition to evaluate the source of the difference at a disaggregated level. The remaining sections of this work are as follows: section two is about the methodology of the study; the third section deals with results and discussion; and the final section is concerned with the conclusion and recommendations.

## 2 Methodology of the study

### 2.1 Type and source of data

The study used secondary data collected in 2017 and 2019 for PSI's Agricultural Growth Program II baseline study and midline evaluation. This data is multi-topic and collected from representative households across the high-potential woreda in the country. This data contains detailed agricultural production parameters covering the size of land, characteristics of land, crop production, crop commercialization, agricultural production and technologies, livestock, nonfarm activities, crop harvest, extension, and access to infrastructure. The AGPII baseline data includes both the treatment and control groups, whereas the AGP-II midline data only includes the treated group. Only 2200 households of the baseline sample was used to gather the midline data. For this study, the researcher utilized the panel households on both the baseline and midline surveys since the purpose of the paper is to compare the gender productivity gap between male-headed households and female-headed households that benefit equally from the AGP II program. Thus, the 926-panel households were dropped from the analysis because of the control group and matching problem, and only 3474 households were used for the analysis, of which 69% (2404) were male-headed households and 31% (1070) were identified as female-headed households.

### 2.2 Method of data analysis

Before detailed productivity gap determination, crop productivity was measured using two methods. The first is an output-area-based estimation by dividing output produced per hectare of land. It was calculated as follows:

(1) $${YI}_{i}=\sum_{i=1}^{n}y_{ij}*\frac{a_{ij}}{A_{ij}}$$

Where *Y*_*i*_ is the overall yield of household *i*, and *i* = 1, 2, …, *n*; *y*_*ij*_ is the per hectare yield of crop *j*, and *j* = 1, 2, …, *M* in household; *a*_*ij*_ is the area of crop *j* of household *i*; and, *A*_*ij*_ is the total cropping area of household*i*.

The second method was calculated by dividing the output value by the input cost per hectare. It has been calculated as follows:

(2) $$Yi=\frac{\;\sum_{i=1}^{N}P_{ij}Q_{ij}}{\sum_{i=1}^{N}C_{ij}X_{ij}}$$

Where, *Y*_*i*_ is the overall yield of household *i*, and *i* = 1, 2, …, *n*; p_ij_ is the price of per hectare output of crop j for ith household head, Q_ij_ is the quantity of output produced for crop j of household I, C_ij_ is the cost of input j for ith household and X_ij_ is the quantity of input j for an ith household.

The traditional approach to measuring and modeling disparities in technical efficiency between men and women in agricultural productivity involves the estimation of production functions that model the greatest output produced from the set of inputs given the technology accessible to the household [6]. The gender gap has been studied in the literature using different approaches. Some research has focused on differences in resource endowments to explain this phenomenon. A common method in this literature consists of testing the allocative efficiency of the distribution of certain inputs such as fertilizer or pesticide between male-headed households and female-headed households by regressing these inputs against households' observable characteristics and a variable identifying the head's gender. These inputs are considered inefficiently allocated assuming decreasing marginal returns when the coefficient of the gender variable is statistically significant. On the other hand, several other studies have tried to explain the gender productivity gap through differences in technical efficiency. Most of these studies regress the yields on the observable characteristics of a pooled sample of male-headed households and female-headed households by including the gender of the head, which accounts for the differences in technical efficiency. Following Ali et al. [4], before looking into the elements that can contribute to potential male-female productivity differences, the study first assessed the existence and size of the gender productivity gap using a standard methodology in the empirical literature.

(3) $$Y_{ih}={\beta Z}_{ih}+{\gamma G}_{ih}+\mu_{ih}+\in_{ih}$$

where Y_ih_ is the log of yield (crop output per planted area) or value of yield for *i*th household *h* ***β****Z*_*i*ℎ_ is the vector of covariates including land characteristics, log of agricultural input per planted area (chemical, organic, labor, etc.), crop varieties and farmer characteristics, γG_ih_ is the binary variables capturing the gender of household head, μ_ih_ is fixed effect that captures all time invariants characteristics of the household. This model extended to panel data and crop-fixed effects. The DID model of impact evaluation was then used to capture the gender gap in agricultural productivity. Difference-in-differences (DiD) is one of the most frequently used methods in impact evaluation studies. Based on a combination of before-after and treatment-control group comparisons, the method has an intuitive appeal and has been widely used in economics, public policy, health research, management, and other fields[31]. A DiD estimate of reform impact can then be constructed, which in its simplest form is equivalent to calculating the after-before difference in outcomes in the treatment group and subtracting from this difference the after-before difference in the control group.

Each model's specification details are listed below, one by one. The difference-in-differences (DiD) design takes into account any difference between genders over a given period. Following this [31], the impact analysis was maintained as follows:

(4) $$DiD=\left(\left({\bar{\text{y}}}_{\text{s}}={\text{treatment}}_{,\;\text{t}=\text{After}}-{\bar{\text{y}}}_{\text{s}}={\text{treatment}}_{,\;\text{t}=\text{Before}}\right)-\left({\bar{\text{y}}}_{\text{s}}={\text{Control}}_{,\;\text{t}=\text{After}}-{\bar{\text{y}}}_{\text{s}}={\text{Control}}_{,\;\text{t}=\text{Before}}\right)\right)$$

Where y is the outcome variable, the bar represents the average value (averaged over households, typically indexed by i), the group is indexed by s (because in many studies, policies are implemented at the household level), and t is time. Algebraically, the equation of DID is represented as follows:

(5) $$y_{\text{it}}=\alpha_{T}+\beta X_{\text{it}}+\mu_{i}+\varepsilon_{\text{it}}$$

where *Y*_*it*_ is the program outcome of interest (yield, revenue, etc.) for household i at time t, T is a dummy variable taking the value one for the year of post-intervention and zero otherwise, *X* is a vector of exogenous variables including household characteristics that may affect the outcome of interest, α and β (a vector) are population parameters to be estimated, ***μ***_*i*_ denotes time-invariant unobserved household-specific heterogeneities, and ***ε***_*it*_ denotes an error term that is assumed to be identically and independently distributed. Our variable of interest is T which shows the average gender yield gap given *y*_*it*_. The before-and-after comparison assumes that if the program had never existed, the program development outcome (yield, revenue) for program participants would have been the same as their preprogram situation. In addition to capturing the unobserved heterogeneity between female-headed households and male-headed households, gender productivity gap determinants were analyzed using the random effect Tobit regression using household panel data. A regression model with panel-level random effects specified as follows:

$$y_{it}=x_{it}\;\beta {+}_{i}+\varepsilon_{it}\;\text{Where}$$

*i* = 1. *n* Panels and *t* = 1 … *n*_*i*_ the random effect *v*_*i*_ are i.i.d. N(0, $\sigma_{\varepsilon }^{2}$ v and ***ε***_*it*_ are i.i.d N(0, $\sigma_{\varepsilon }^{2}$ σ)independently of *v*_*i*_. The observed data represent possibly censored versions of *yit*.

Furthermore, in the literature, different methodologies have been used by Ali et al. [4], Bonis-Profumo et al. [27], Bello et al. [11], Pierotti et al. [32], Britos et al. [33], Maisonnave and Mamboundou [34] to examine the gender gap in agricultural productivity between male-headed households and female-headed households. This analysis also follows a similar approach to the previous empirical works (i.e., [1,2,6,10,12]) and employs the extended Oaxaca-Blinder decomposition technique [18,19]. The standard Oaxaca decomposition [18] used in this analysis and the equation that has been used to decompose is as follows:

(6) $$Q_{m}‐Q_{f}=b_{m}\left(X_{m}‐X_{f}\right)+\left(b_{m}‐b_{f}\right)X_{f}$$

WhereQ_m_, and Q_f_, represents the mean yields of male-headed households and female-headed households respectively. b_m_, and b_f_, are estimated output coefficients of male-headed households and female-headed households farmers, and XM, and *Xf*, are mean levels of endowments and inputs of male-headed households and female-headed households farmers. That is, the overall average male-female yield gap can be decomposed into the portion due to differences in input endowments, (*X*_*m*_ − *X*_*f*_) evaluated using male coefficients; the other portion is attributable to differences in the returns, or output elasticity (*b*_*m*_ − *b*_*f*_), that male-headed households and female-headed households get for the same endowment or input application lastly, to overcome the weakness of [18] the authors use the RIF decomposition method [24]. RIF's decomposition is an improved extension and refinement of the standard Oaxaca–Blinder [18] decomposition techniques. RIF provides the detailed contributions of individual covariates to aggregate decomposition [24]. Following Rios Avila [24], and Tufa et al. [10] the researchers assume that *f*_*y*, *x*, *g*_ (*Y*_*i*_*X*_*i*_*G*_*i*_ is a joint distribution function that describes all relationships between household productivity (y) and head of the household characteristics X; as well as the sex of the household head. The joint probability distribution function and cumulative distribution of Y conditional on (G) can be expressed as:

(7) $$f_{Y,X}^{g}\;\left(Y,X\right)=f_{Y|X}^{g}\left(Y|X\right)\;f_{x}^{g}X$$

(5) $$f_{Y,}^{g}\left(y\right)=\int f_{Y|X}^{g}\left(Y|X\right)d\;f_{x^{X}}^{g}$$

Where superscript g indicates the density of conditional on G = g with gϵ[0,1]. To analyze the difference in productivity between male head households (g = 0) and female head households (g = 1) for a given distributional statistics v the cumulative conditional distribution of Y can be used to calculate the agricultural productivity gap.

(8) $$\Delta v=v_{1-}v_{0}=v\left(F_{Y}^{1}\right)-v\left(F_{Y}^{0}\right)$$

(9) $$\Delta v=v(f_{Y,}^{g}\left(y\right)=V(\int F_{Y|X}^{1}\left(Y|X\right)d\;F_{x}^{1}-vV(\int F_{Y|X}^{0}\left(Y|X\right)d\;F_{x}^{0}X$$

Equation (2) shows that the difference in the statistics Δ*v* arises from the difference in the distribution of Xs (*d F*_*x*_^1^(*X*) ≠ *d F*_*x*_^0^(*X*)) and the difference in relationships between Y and

($F_{Y/X}^{1}\left(Y/X\right)d\;F_{x}^{1}\neq F_{Y/X}^{0}\left(Y/X\right)d\;F_{x}^{0}X$. To decompose the overall productivity gap caused by the structural effect the researcher obtains the counterfactual using the VC [24].

(10) $$v_{c}=v\left(F_{Y}^{c}\right)=v\left(\int F_{Y|X}^{0}\left(Y|X\right)d\;F_{x}^{1}\left(X\right)\right)\;$$

The gap in distribution statistic v can be disaggregated into two effects: the endowment Δ*v*_*x*_ and structural Δ*v*_*s*_ effects, as follows:

(11) $$\Delta v=\left(v_{1}-v_{c}\right)+\left(v_{c}-v_{0}\right)$$

We use the semi-parametric reweighting p procedure to identify the counterfactual distribution $F_{Y|X}^{0}\left(Y|X\right)d\;F_{x}^{1}$ (X) based on the observed data. According to Rios Avila [24], although we cannot directly observe the distribution of outcomes and characteristics, the researchers can approximate the counterfactual distribution by multiplying the observed distribution of characteristics *d F*_*x*_^0^(*X*) with a factor ***ω***(*X*) thus representing the distribution *F*_*x*_^1^(*X*). Therefore, the counterfactual function in the equation can be rewritten as:

(12) $$F_{Y}^{c}=\int F_{Y|X}^{0}\left(Y|X\right)d\;F_{x}^{1}\left(X\right)≅\int F_{Y|X}^{0}\left(Y|X\right)d\;F_{x}^{0}\omega \left(X\right)$$

The reweighting factor ω(X) can be identified using the Bayes rule as follows:

(13) $$\omega \left(X\right)=\frac{d\;F_{X}^{1}\left(X\right)}{d\;F_{X}^{0}\left(X\right)}=\frac{\;{\text{dF}}_{X|G}\left(X|G=1\right)}{\;{\text{dF}}_{X|G}\left(X|G=0\right)}=\frac{{\text{dF}}_{X|G}\left(X|G=1\right)}{{\text{dF}}_{G}\left(G=1\right)}=\frac{{\text{dF}}_{G}\left(G=0\right)}{{\text{dF}}_{G|X}\left(G=0|X\right)}=\frac{1-P}{P}\;\frac{P(T=1|X}{1-P(T=1|X}$$

Where p is the proportion of the head of the household in group *G* = 1 and *P* (*G* = 1|*X*) is the conditional probability of the household head with characteristics X being part of group 1. This implies the counterfactual distribution of the *F*_*Y*_^*c*^_|*X*_, can be identified by estimating the reweighting factor (*X*) using the parametric methods to estimate the conditional probability of *P* (*G* = 1|*X*). After reweighting the factors of counterfactual statistics *v*_*c*_ the researcher estimates RIF regression for each group and counterfactual as follows:

(14) $$v_{1}=E\left(\text{RIF}\left(y_{i},v\left(F_{Y}^{1}\right))\right)={\bar{X}}^{1^{\prime }}\;\beta^{1}\right)$$

(15) $$v_{0}=E\left(\text{RIF}\left(y_{i},v\left(F_{Y}^{0}\right))\right)={\bar{X}}^{0^{\prime }}\;\beta^{0}\right)$$

(16) $$v_{c}=E\left(\text{RIF}\left(y_{i},v\left(F_{Y}^{0}\right))\right)={\bar{X}}^{c^{\prime }}\;\beta^{c}\right)$$

Therefore, after some mathematical tricks, the final decomposition components were defined as

Follows:

(17) $$\Delta v=\underset{{\Delta v}_{x}^{p}}{\underbrace{{{\left({\bar{X}}^{c}-{\bar{X}}^{m}\right)}^{\prime }}^{\beta_{m}}}}+{\bar{X}}^{c}\underset{{\Delta v}_{x}^{e}}{\underbrace{\left({\overset{´}{\beta }}_{c}-{\overset{´}{\beta }}_{m}\right)}}+\underset{{\Delta v}_{s}^{p}}{{\bar{X}}^{f^{\prime }}\underbrace{\left({\overset{´}{\beta }}_{f}-{\overset{´}{\beta }}_{c}\right)}}+\underset{{\Delta v}_{s}^{e}}{\underbrace{{\left({\bar{X}}^{f^{\prime }}-{\bar{X}}^{c}\right)}^{\prime }{\overset{´}{\beta }}_{c}}}$$

The components ${\Delta v}_{x}^{p}$ + ${\Delta v}_{x}^{e}$ resemble the Oaxaca-Blinder aggregate endowment effect and ${\Delta v}_{s}^{p}$ +${\Delta v}_{s}^{e}$ resemble the aggregate structural effect. ${\Delta v}_{x}^{p}$ and ${\Delta v}_{s}^{p}$ represent pure endowment and structural effect. ${\Delta v}_{s}^{e}$ and ${\Delta v}_{x}^{e}$ asses the overall fitness of the model.

A clean version of the data has been prepared primarily before doing the analysis. To determine the productivity gap between male- and female-headed households over different periods and groups, the DID model was employed. Then, the random effect Tobit model was used to determine the determinants of productivity differences among female and male-headed households. Finally, the Oaxaca Blinder decomposition and the RIF's Oaxaca decomposition were used to identify the magnitude and source of the gender productivity differential.

### 2.3 Variables of interest and definition

The variables of interest, together with their definition and expected sign of influence, were identified by the researchers. The summary of variables of interest definition kept below (See Table 1).

**Table 1:** Variables of interest and definition.

Variables of Interest	Definition of Variables	Exp. Sign
**Dependent variable** Log of crop productivity	The logarithm of crop productivity, the ratio of gross output divided by area in hectares, and gross output value divided by input value	
**Independent Variables** Age of household head	Age of a farmer in years	–
Marital status	Marital status of a farmer (Married = 1 and single = 0)	+
Sex of the household head	Sex of the farmer (male = 1 and female = 0)	+
Educational level	Education level of the farmers in years	+
Family size	Family size	+
Livestock ownership	Livestock owned measured by TLU	+
Access to credit	Farmers' access to credit (1 if has access to credit and 0 otherwise)	+
Access to extension service	Access to extension service (1 if farmer has access and 0 otherwise)	+
Use of chemical fertilizer	Use of chemical fertilizer (1 if the farmer has used it and 0 otherwise)	+
Farm size	Area of farmland (hectares)	–
Market access	Access to market (1 if farmer has access and 0 otherwise)	+
Soil fertility status	Takes 1 if a farmer perceives that the soil is fertile and 0 otherwise	+
Irrigation access	Access to irrigation (1 if the farmer has access and 0 otherwise)	+
Topography of plot	Takes 1 if a farmer perceives that the plot is flat and 0 otherwise	+
Average distance to plots	Distance from home to a plot in walking distance in a minute	–
Multiple crops produced	Number of crop types produced by a farmer (1 if the farmer produced one crop and 0 otherwise)	–
Planting method	Takes 1 if a farmer used row planting and 0 otherwise	+
Mechanization	Takes 1 if a farmer used mechanization and 0 otherwise	+

## 3 Result and discussion

### 3.1 Sample households’ characteristics

In this section, various continuous socio-demographic and socio-economic variables were tested for significant mean differences among male and female household heads included in the sample. Accordingly, the major significant variables are presented as follows:

The mean education level of the respondents was 2.36, showing the lower education level of farm households. There was a positive and significant relationship of 1% significance between the sex of the household head and education level. The female household head group (n = 1070) has a mean education level of 2.12 (SD = 3.16). By comparison, the male group was associated with a numerically higher education level of 2.46 (SD = 3.5). Thus, male household heads were associated with statistically higher mean education levels (Table 2).

**Table 2:** Descriptive result for continuous variables.

Variables	Male (N = 2404)		Female (N = 1070)		Total sample (N = 3474)		t-value
	Mean	St. Dev.	Mean	St. Dev	Mean	St. Dev	
Age	45.650	14.23	45.87	14.38	45.72	14.27	0.42
Education (years)	2.468	3.53	2.12	3.17	2.36	3.43	23.71***
Land size (ha)	1.621	1.56	1.60	1.42	1.62	1.52	0.32
Family size	5.584	2.09	5.50	2.18	5.56	2.11	1.04
Livestock owned	3.850	1.39	2.81	1.36	3.84	1.38	4.55**

The mean livestock ownership measured in Tropical Livestock Units (TLU) was 3.84. Female-headed households have a mean livestock ownership of 2.81, while male-headed households have a mean of 3.85. Thus, there is a significant livestock ownership difference between male-headed and female-headed households at a 5% significance level (Table 2). As most of the land preparation is done by animal draught power, the contribution of livestock ownership to production and productivity is unquestionable. Moreover, ownership of livestock increases the probability of using manure in crop production, which would contribute to soil fertility improvement. Since land preparation and soil fertility management practices' contribution to crop productivity is high, livestock ownership is expected to have a significant impact on the gender agricultural productivity gap.

The sample was composed of both male- and female-headed households. From the total sample respondents, 2404 (69%) were male household heads and 1071 (31%) were female household heads. Most of the sample household heads were married, and of these married household heads, 69% were male and 31% were female. However, the difference in terms of marital status between the two groups was not statistically significant (Table 3).

**Table 3:** Summary of a categorical variable group of female and male household heads.

Variables	Female	Male	Pearson χ 2
	N = 1070)	(N = 2404)	
Married household heads (Yes %)	30.70	69.30	0.023
Perceived fertile soil (Yes %)	65 .00	66 .00	2.130**
Perceived flat plot topography	80.00	78.00	1.530
Grow multiple crops (Yes %)	53.00	48.00	0.410
Used improved seed and row planting method (Yes %)	12	15	11.04***
Have extension contacts (Yes %)	24	65	1.760
Used machine harvesting (Yes %)	7	9	2.680*
Have irrigation access (Yes %)	13	13	0.200
Have credit access (Yes %)	47	45	0.110
Have market access (Yes %)	25	25	0.800
Used fertilizer on crops (Yes %)	67	68	4.080***

From the total sample of households, 78.64% perceived that their land was fertile soil, and the remaining perceived it was not. The difference in terms of soil fertility and the status of plots among the two groups was significant at a 5% level of significance (Table 3). The possible reason could be that most soil and water conservation activities with soil fertility management are primarily practiced by the male household head, and the use of chemical fertilizer by women farmers is affected by various socio-economic statuses. As shown in the table below, the difference in terms of the use of chemical fertilizer among the two male and female household heads was significant at a 1% level of significance.

Despite the importance of appropriate agricultural technologies, such as improved seed, for improving the productivity of crop production, the use of improved seed with row planting and the application of mechanization is low among farmers. The proportion of female-headed households using improved seed and planting methods was 12% significantly lower than that of 15% of male-headed households. From this, the contribution of female household heads was minimal, showing significant differences between the two groups in the use of improved seed and row planting. Similarly, there is a significant difference between male and female household heads in the use of mechanization, especially machine use for crop harvesting. The proportion of female-headed households using machine harvesting was 7%, significantly lower than that of 9% of male-headed households (shown in Table 3 above).

Generally, the descriptive comparison revealed that female-headed households are significantly cultivating less fertile land, have weak use of improved seeds and row planting methods, poorly apply chemical fertilizers, lack credit access, and are more likely to be using mechanization technologies than male-headed households.

### 3.2 Crops production and productivity difference among male and female-headed households

Major cereal crops produced were maize, teff, wheat, sorghum, barley, and wheat, based on their area coverage and contribution to producers' food security and economy. The mean area covered by cereal crops of the total sample households was 0.76 ha. There was a positive and significant relationship between the sex of the household head and cultivated land coverage at a 5% significance level. The female-headed households have a mean of 0.69 ha, which is lower than the males (0.75 ha) (Table 4 below). The mean harvested of male and female-headed households is statistically and significantly different at a 5% significance level. Female-headed households harvested a lower mean harvest (10.05 q/l) than males of 12.27 q/l in a production season. There was a statistically significant difference in the production and harvest of crops between the two groups. The lower harvest and comparatively higher input costs of female-headed households contributed to lower output values compared to male-headed households.

**Table 4:** Crops production and productivity difference among male and female-headed households.

Variables	Male (N = 2404)		Female (N = 1070)		Total sample (N = 3474)		t-value
	Mean	SD	Mean	SD	Mean	SD	
Crops area	0.75	0.75	0.71	0.66	0.74	0.72	2.213**
Production (ql)	12.26	19.51	10.05	12.56	11.58	17.69	4.00***
Input cost (2USD)	289.45	559.38	263.94	336.18	281.59	501.45	1.67*
Output value (USD^3^)	406.44	813.62	358.92	530.59	391.80	738.36	2.05**
Log Productivity (value-based)	1.64	1.03	1.46	0.83	1.58	0.97	4.93***
Log Productivity (area-based)	16.26	11.92	15.04	9.91	15.88	11.35	3.15***
Average distance to plots (minutes)	16.22	17.57	16.22	16.56	16.27	17.26	0.10

2,3 The output value and input cost was measured by Ethiopian Birr at Survey time and then converted to United State dollar by using average annual exchange rate of the year 2019.

This can be revealed by the fact that male household heads earned on average 406.44 US dollar in a production season, which is higher than female-headed households of 358.92 USD, and the difference was statistically significant at a 5% significance level. In this study, crop productivity has been measured in both output and input costs and output and area coverage. In both estimation methods, female-headed households showed lower productivity levels than male-headed households. For instance, male household head output-area-based productivity on average was 16.26 q/l per ha in the main production season, which is higher than female-headed households of 15.04 q/l, and the difference in crop productivity between the two groups is statistically significant at the 1% significance level (Table 4 above).

### 3.3 Productivity improvement based on DiD measurements

The difference in difference estimation strategy was used to calculate the gender productivity gap between male and female-headed households in the years 2016/2017 and 2019, following the AGPII baseline data and the AGPII midline data.

As presented in Table 5, female-headed household productivity improved by 0.22, measured by the output-input method, and by 0.55 quintals based on the output-area-based measurement. The male-headed household productivity improved by 0.26, measured by the output-input method, and by 2.6 quintals based on the output-area-based measurement. The difference in difference measured for the two groups was 4% measured by the output-input method and 2.05 quintals based on the output-area-based measurement. This result showed improvement in both groups due to AGP program intervention; however, male-headed households' improvement has been considerably higher than female-headed households.

**Table 5:** Productivity improvement based on DID.

Year	Sex of household head	Log productivity is measured in value	Log Productivity measured in area-based
**2017**	Female	1.36	14.78
	Male	1.52	15.05
**2019**	Female	1.58	15.33
	Male	1.78	17.65
	Female difference	0.22	0.55
	Male difference	0.26	2.60
	Difference in difference	0.04	2.05

### 3.4 Determinants of household heads' agricultural productivity

Factors affecting household head productivity were further examined through the Tobit regression model. For both value-based and area-based regressions, various sets of explanatory variables were found to be significant. The sex of the household head, soil fertility status, extension contact, and credit use were found to have a positive and significant effect on productivity. A flat topography, more livestock ownership, the use of improved seed and row planting, and the use of chemical fertilizer, and machine for harvesting affected productivity positively and significantly. The researchers estimated the random effect Tobit model, which strengthens the result from the pooled Tobit model. The output from the random effect Tobit model includes the overall and panel-level variance components rho, and we have tested the difference between the two models using the likelihood-ratio test, which is included at the bottom of the output. When rho is zero, the panel-level variance component is unimportant, and the panel estimator is not different from the pooled estimator. In our case, we reject the null hypothesis that there are no panel-level effects and go for the random effect, and the random effects model is calculated using quadrature, which is an approximation whose accuracy depends partially on the number of integration points used. We checked this using the quadchk command to see if changing the number of integration points affected the results, and we confirmed no impact. The results of the random effect show that, given the unobserved heterogeneity under the random effect Tobit model, unobserved household characteristics are the cause of the gender gap in agricultural productivity.

Among the observed variables, soil fertility and extension contact are the main factors accounting for the gender gap in agricultural productivity in all scenarios under consideration (See Table 6). Credit use and family size showed an unexpected negative sign. However, it might be because household heads with larger families borrowed and utilized their credit for consumption smoothing rather than using it for applying productivity-improving technologies such as improved seed, fertilizer, and others. The finding is consistent with some prior findings that revealed the main causes of the gender gap, pinpointed as gender differences in access to and use of agricultural inputs and related investments in land and improved technologies, market and credit access, human and physical capital, and informal institutional constraints affecting farm management and marketing of agricultural outputs. For instance, compared to male-headed households, plots managed by women were found to be on average 37.5% less productive in Malawi [10], 34.9% less productive in Uganda [4], 25% less productive in Malawi [8], 23.4% less productive in Ethiopia [1], 20% less productive in Mozambique [15], and 11% less productive in Nigeria [11]. The main causes of the gender gap in these studies have been pinpointed as gender differences in access to and use of agricultural inputs, tenure security, and related investments in land and improved technologies; market and credit access; human and physical capital; and informal institutional constraints affecting farm and plot management and marketing of agricultural produce.

**Table 6:** Pooled and Random Effect Tobit model result on the Determinants of the gender gap on productivity.

Variables	Productivity (value-based)						Productivity (area based)					
	Pooled Tobit Model Result			Random Effect Tobit Model Result			Pooled Tobit Model				Random Effect Tobit Model Result	
	Coeff.	Margin al effect	z-value	Coef f.	Margi no effect	z-value	Co eff.	Margin al effect	z-value	Coeff.	Marginal effect	z-value
Age	0.000	0.000	−0.240	0.000	−0.000	−0.010	0.000	0.001	0.710	0.001	0.001	0.860
Sex of household head	0.094	0.095	6.170***	0.097	0.097	6.380***	0.040	0.044	1.770*	0.046	0.046	1.850*
Education	0.003	0.003	1.240	0.002	0.002	1.010	0.003	0.003	0.860	0.003	0.003	0.730
Marriage	0.015	0.015	0.830	0.014	0.014	0.800	0.036	0.036	1.210	0.036	0.036	1.200
Soil fertility	0.041	0.041	2.640***	0.033	0.033	2.190**	0.134	0.134	5.310***	0.128	0.128	5.070***
Slope	0.051	0.051	2.870***	0.042	0.042	2.410**	0.013	0.012	0.420	0.004	0.004	0.130
Av. distance to the plots	0.001	0.000	−1.100	0.000	0.000	0.250	−0.000	−0.001	−0.820	0.000	−0.000	−0.030
Livestock holding unit(TLU)	0.014	0.014	2.780***	0.007	0.007	1.360	0.004	0.004	0.450	−.003	−0.003	−0.360
Family size	0.001	0.001	0.200	0.001	0.001	0.380	−0.011	−0.011	−1.970**	−0.010	−0.010	−1.880**
Land size	0.002	0.002	0.390	0.000	0.000	0.050	−0.011	−0.011	−1.340	−0.012	−0.012	−1.540
Growing multiple crops	−0.019	−0.019	−1.270	−0.035	−0.035	−2.430**	0.016	0.015	0.650	0 .000	−0.000	−0.020
Extension contacts	0.049	0.049	3.350***	0.042	0.042	2.940***	0.104	0.104	4.330***	0.098	0.098	4.090***
Plant method(improved Seed)	0.056	0.056	3.710***	0.026	0.026	1.680	0.032	0.032	1.280	0.003	0.003	0.100
Mechanization	0.027	0.027	1.00	*.*032	0.032	1.220	0.161	0.1607	3.63***	.166	0.166	3.76***
Fertilizer use	0.098	0.098	6.93***	.105	0.105	7.510***	0.024	0.0235	1.02	.03	0.030	1.29
Irrigation access	−0.002	−0.002	−0.08	.004	0.004	0.170	0.039	0.0392	1.12	.044	0.044	1.26
Credit use	0.051	0.051	3.56***	*.*008	0.008	0.560	−0.089	−0.0885	3.76***	−.128	−0.128	−5.23***
Market access	0.021	0.021	1.30	*−*.004	−0.004	−0.280	0.042	0.0424	1.58	.018	0.018	0.65
Constant	0.028		0.57	.1	1.35		2.375		29.52	2.441		25.17
	Mean dependent var = 0.331 SD dependent var = 0.471 Pseudo r-squared = 0.044 Number of obs = 3269 Chi-square = 173.991 Prob > chi2 = 0.000 Akaike crit. (AIC) = 3863.628 Bayesian crit. (BIC) = 3985.473			/sigma_u = 0.050 /sigma_e = 0.00 Rho = 0.03 LR test of sigma_u = 0: chibar2(01) = 90.14 Prob ≥ chibar2 = 0.000			Mean dependent var = 2.559 SD dependent var = 0.673 Pseudo r-squared = 0.016 Number of obs = 3269 Chi-square = 106.176 Prob > chi2 = 0.000 Akaike crit. (AIC) = 6621.374 Bayesian crit. (BIC) = 6743.219				/sigma_u = 0.059 /sigma_e = 0.00 Rho = 0.013 LR test of sigma_u = 0: chibar2(01) = 27.14 Prob ≥ chibar2 = 0.000	

***p < .01, **p < .05, *p < .1.

### 3.5 Oaxaca Blinder decomposition for the gender gap in crop productivity

The study found a modest difference in crop productivity between female-headed and male-headed households of 11.1%(See Table 7 below). However, the productivity difference is lowering up to 5% when measured through the output to cropping area. After accounting for observed household head characteristics, the unexplained difference in yield between male and female-headed households is 10.5% and 4.9% See Table 7 below) in output value to input cost conversion and output to area productivity measurement, respectively. Female-headed households cultivate smaller areas of crop production in a context with strong inverse returns to the planted area, giving them a net endowment advantage of 0.6%. Crop yields in male-headed households are about 4.9–10.5% more productive than yields on farms in female-headed households. The structural component of the gap is larger than the endowment component, suggesting that even if women possess the same characteristics as men and are provided equal access to productive resources, the productivity difference will continue unless the returns to the resource endowed improve. Some prior studies similarly revealed that the gender productivity gap is driven majorly by differences in returns to resource endowment or structural effects between male- and female-headed households (for instance, Tufa et al. [10] in Malawi, Ali et al. [4] in Uganda, and Bello et al. [11] in Nigeria). Female-headed households’ showed a structural disadvantage and an endowment advantage. For instance, Tufa et al. [10] showed females a structural disadvantage of 23.1% and an endowment advantage of 8.2%); Ali et al. [4] revealed an unexplained yield difference between male and female farmers to be 30.4% after taking into account observed parcel characteristics and unobserved household, community, season, and farmer factors,

**Table 7:** Log Value of productivity Oaxaca Decomposition by Gender of household heads.

Aggregate decomposition	Log productivity by value decomposition		Log productivity by area-based decomposition	
	Coefficient	P-value	Coefficient	P-value
Male-headed households' productivity(log)	0.365	0.000	2.575	0.000
Female-headed households' productivity(log)	0.255	0.000	2.525	0.000
Difference in productivity	−0.111	0.000	−0.050	0.046
Explained Portion of Difference	−0.006	0.247	−0.001	0.886
Unexplained Portion of Difference	−0.105	0.000	−0.049	0.049

Further analysis by the Oaxaca-Blinder decomposition method revealed that some factors contributing to the female household head productivity gap were livestock ownership, the use of improved seed and row planting methods, and fertilizer application. See Table 7 below.

#### 3.5.1 Detail decomposition based on value-based productivity measurement

The RIF Oaxaca approach is used to decompose the productivity gap between male and female-headed households into a component that can be explained by variations in productivity determinants and a part that cannot be explained by such group differences. The results of RIF decomposition are shown in the table below. The major findings revealed that households with female heads were, on average, 11.1% less productive than households with male heads. The Oaxaca decomposition result revealed that the method of applying fertilizer, the number of livestock held, and the usage of machinery were the factors that contributed to the lower production of families with female heads. Access to credit, TLU, extension, planting method, fertilizer use, soil fertility, and slope, in contrast, make the male-headed household more productive.

From Table 8 above, one can understand that Access to agricultural extension services, the technique of applying fertilizer, the number of livestock held, and the usage of machinery were the factors that contributed to widening the gap, suggesting that less attention may be given to women in agricultural extension programs, technology adoption, and asset holding. Lad size, multiple crop planting, and irrigation access are factors that close the gender productivity gap, even though they are not statistically significant. Beyond this, access to all other socioeconomic characteristics contributes to the gender gap, even if they are not statistically significant. For the male-headed household, soil fertility, the slope of the land, extension, planting method, technique of fertilizer application, and credit access contribute 34% of gender differentials, whereas, for the female-headed household, fertilizer application plays the major role in gender differentials, followed by planting method and total livestock unit.

**Table 8:** Detail decomposition based on Value Based Productivity measurement.

Variables	RIF regression group 1(Female)		RIF regression group 1(Male)	
	Coef.	z-value	Coef.	z-value
Age	−0.001	−0.840	0.000	0.000
Education	0.004	0.830	0.001	0.460
Marriage	0.050	1.250	0.001	0.030
Soil fertility	0.047	1.410	0.042	2.050***
slope	0.062	1.560	0.062	2.660***
distance	0.001	0.550	−0.001	−1.500
TLU	0.026	2.270**	0.012	1.730*
Family size	0.006	0.800	0.002	0.340
Land size	0.012	1.080	−0.001	−0.180
Multiple crops	−0.055	−1.730*	−0.019	−0.990
extension	0.018	0.540	0.056	2.890***
Planting method	0.094	2.790***	0.048	2.370***
Mechanization use	0.042	0.740	0.044	1.190
Fertilizer application	0.160	4.870***	0.100	5.470***
Irrigation access	0.034	0.740	−0.002	−0.070
Credit access	0.008	0.260	0.059	3.070***
Market access	0.015	0.420	0.019	0.870
_cons	−0.122	−1.130	0.112	1.770
	RIF mean: 0.3653		RIF means: .25471	
	Number Of obs = 1,019		Number Of Obs. = 2250	
	Prob > F = 0.000		Prob > F = 0.000	
	R-squared = 0.055		R-squared = 0.037	

#### 3.5.2 Detail decomposition area-based productivity measurement

The productivity gap between households with male and female heads was calculated using the area-weighted productivity. According to the findings, households headed by women are 5% less productive than households headed by men. Similar to the last instance, the use of mechanization, soil extension, and soil fertility were all factors in the production differential. On the other hand, factors like soil fertility, marriage, credit access, market access, extension, planting method, mechanization use, and fertilizer application method all helped male-headed households produce more. Family size, however, has a detrimental impact on the productivity of male-headed households since the larger the family, the more they exploit their land using family labor and the less productive the land is. Therefore, farms run by households with male heads produce around 11.2% more than farms run by households with female heads. The finding is consistent with other prior studies that found nearly the same productivity gap among male and female households 4–40% in Malawi [8]; 11% in Nigeria [11,35]. However, the gender productivity gap estimated is lower than some other studies that revealed less productivity of women with a gap of an average of 37.5% in Malawi [10], 34.9% in Uganda [4], 25% in Malawi [8], 23.4% in Ethiopia [1], 20% and in Mozambique [15]. The major reason for that might be due to some policy and project interventions that contributed to women's empowerment and female-headed households' productivity improvements in the last years including the AGP program, for instance.

The RIF Oaxaca decomposition reveals that the structural effect outweighs the endowment effect in terms of the percentage of gaps attributable to variations in observed household factors. As a result, the structural component of the difference is greater than the endowment component. According to this finding, performance gaps will still exist even if women share the same traits as men and have equal access to both productive resources and policy variables. The above result reveals that given equal access to resources, there is still a difference between male-headed and female-headed households, where soil fertility, extension service, and mechanization use together contribute 48% to gender differentials. From Table 9 above, one can simply trace that the male-headed household has more access to the endowment, which helps strengthen the structural characteristics that result in gender differentials. Prior studies also explored the relevant contributions of the resource endowment effect versus the returns to resources and structural effects of the gender gap in productivity. Some of the author's conclusions have been that the gap is driven by differences in resource endowment between male- and female-headed households, rather than returns to resource endowment. For instance, Aguilar et al. [1] in Ethiopia, Kilic et al. [8] in Malawi, and Makate and Mutenje [16] in Tanzania showed 13.4%, 82%, and 70.3%, of this difference in productivity gap is explained by observable characteristics, or resource endowment respectively. Many of these studies suggest that estimates of the gender productivity gap become smaller after disparities in access to productive resources and personal traits are taken into account. Others revealed that the gap is driven primarily by differences in returns to resource endowment and structural effects between male- and female-headed households. For instance, Tufa et al. [10] in Malawi, Ali et al. [4] in Uganda, and Bello et al. [11] in Nigeria showed that 23.1%, 30.4%, and 77.6% of the difference in productivity gap are explained by unobservable characteristics or returns to resource endowment, respectively. If women's farms have more access to agricultural resources, they may produce 20%–30% more yields [36] compared to what they were producing before. However, other authors argue that the productivity gap would persist even if women shared the same characteristics as men, had equal access to resources for generating income, and were taken into account when making policy decisions [8,11,28]. The gender gap shrinking inside the family may provide women with more power, enhancing their access to productive inputs, increasing their responsibility for decision-making and bargaining power, and enhancing their ability to make their own decisions that are in their economic interest [3,29]. It has been shown that the gender difference in agricultural productivity is related to either the gender of the household head or the gender of the person who manages the farm at home, utilizing data and outcomes at the household level [10]. When compared to men, who have fewer responsibilities at home, women, particularly those who have children, spend less time on farming operations due to concerns about domestic chores, child care, and other duties. Women are time-constrained in rural communities because they manage the household and care for the children full-time. This is especially true when it comes to attending training for the extension service and taking care of domestic duties at the same time. Lack of extension results in the inability to adopt new technology in farming. Consequently, farm production may decline, and farm output may be lower. In addition, a positive coefficient for the extension variable indicates that the variable has contributed favorably to the widening of the gender performance gap. This could be because extension services are not sufficiently addressing the information needs of women.

**Table 9:** Detail decomposition based on area-based approach.

Variables	RIF regression group (Female)		RIF regression group (Male)	
	Coef.	z-value	Coef.	z-value
Age	0.001	0.770	0.000	0.290
Education	−0.004	−0.570	0.005	1.210
Marriage	−0.061	−1.200	0.076	2.060**
Soil fertility	0.139	3.260***	0.133	4.240***
slope	−0.001	−0.020	0.008	0.230
distance	−0.001	−1.110	−0.000	−0.460
TLU	0.018	1.230	−0.003	−0.340
Family size	−0.010	−1.110	−0.013	−1.870*
Land size	−0.023	−1.600	−0.005	−0.510
Multiple crops	−0.039	−0.960	0.036	1.220
extension	0.145	3.470***	0.094	3.180***
Planting method	−0.062	−1.430	0.072	2.370***
Mechanization use	0.199	2.750***	0.144	2.570***
Fertilizer application	−0.063	−1.500	0.055	1.970
Irrigation access	0.034	0.580	0.048	1.100
Credit access	−0.057	−1.410	−0.103	−3.530***
Market access	0.015	0.330	0.055	1.680*
_cons	2.513	18.140	2.391	24.740
	Distributional Statistic: mean		Distributional Statistic: mean	
	Sample Mean RIF mean: 2.5251		Sample Mean RIF mean: 2.5747	
	Number of obs = 1,019		Number of Obs. = 2250	
	Prob > F = 0.000		Prob > F = 0.000	
	R-squared = 0.044		R-squared = 0.037	
	Root MSE = 0.631		Root MSE = 0.676	

## 4 Conclusion and recommendations

Ethiopian agricultural policies have been in place for many years, with an emphasis on increasing agricultural productivity, ensuring food security, and reducing poverty. However, gender disparities that disproportionately disadvantage women are present in the nation's agricultural productivity whereas women comprise more than 50% of the labor force. Investigating the gender gap and factors that contribute to the gender gap literature by providing concrete evidence and quantifies the existing gender productivity gap in Ethiopia's agricultural sector, shedding light on the magnitude of the gap and identifying specific areas and factors contributing to the disparities, identifying the socio-cultural, economic, institutional, and structural factors that contribute to gender disparities in agricultural productivity as well as by identifying the specific areas that need attention, such as access to resources, education, skills development, and market participation for women in agriculture. Consequently, this paper estimates the gender gap in agricultural productivity in Ethiopia and reveals this gap in observed and unobserved household characteristics. This study investigated the gender differences in agricultural productivity and highlighted the main causes of the productivity gaps between male-headed households and female-headed households. By measuring the production differences between male-headed households and female-headed households in Ethiopia.

Employing RIF Oaxaca-Blinder decomposition, the results from the study showed a gender performance (productivity) difference between male-headed and female-headed households of roughly 11.5% when measured by value and 5% when measured by an area-weighted formula.

This decomposition technique allows us to identify and quantify the factors contributing to these differentials, which enables a more nuanced understanding of the underlying dynamics by splitting down gender productivity differentials into two main components: an “explained” part and an “unexplained” part. The explained part refers to the portion of the differential that can be attributed to observable factors such as differences in education, experience, and occupation. On the other hand, the unexplained part represents the residual component that cannot be directly attributed to these observable factors. the RIF technique helps to highlight the relative importance of these different factors in contributing to the overall gaps. The unexplained portion of the differential may indicate the existence of unobserved or unmeasured factors that systematically disadvantage one gender over the other. These unexplained factors could include gender stereotypes and bias in promotions. The RIF's decomposition demonstrated that, although male-headed and female-headed households have equal access to resources, there are still unmeasured differences preventing women from maximizing their resource usage. This shows performance gaps will still exist even if women share the same traits as men and have equal access to both productive resources and policy variables. The above result reveals that given equal access to resources, there is still a difference between male-headed and female-headed households, where soil fertility, extension service, and mechanization use together contribute 48% to gender differentials. The result from RIF decomposition show that soil fertility and extension contact along with technology use are the main contributor to the gender gap in agricultural productivity though an unexplained portion of variation outweigh the explained portion of variation in covariates. The main finding of the study is that endowment effects were less likely to have a significant impact on the productivity gap than the structural effects did. Differences in the unexplained characteristics of men and women may also contribute to the considerable productivity gap between households headed by men and women.

This study shows that to support women's emancipation and address the underlying reasons for gender differences in productivity outcomes, efforts to close the gender productivity gap should go beyond ensuring that everyone has access to resources. Such initiatives might, for example, involve the use of gender transformative strategies such as women-targeted training and field demonstration, Enforcing gender-sensitive labor laws and policies, Implementing gender-responsive social protection programs, Promoting gender-aware economic policies that aim to improve women's negotiation and decision-making abilities while also addressing the gender norms and power dynamics that prevent women from utilizing and benefiting from the resources they have access to. The significance of structural impacts in accounting for the gender productivity gap highlights the need for policies and agricultural development programs that take into account the underlying mechanisms generating gender productivity gaps rather than focusing only on agricultural production aspects.

The findings of this study have significant policy implications for policy targeting. To effectively plan and carry out gender-responsive policies and project interventions, development professionals and policymakers would use the study's findings on the gender gap in agricultural productivity. Consequently, it would be crucial for the Ministry of Women's Affairs to concentrate on women's empowerment to improve their structural disadvantages and increase the returns of resource utilization through various training programs that favor women or gender mainstreamed extension training programs for lowering gender productivity differentials in close collaboration with the Ministry of Agriculture. In addition, the concerned body should work on education and Skills development, Access to finance and entrepreneurship, infrastructure and access to services, social norms, and gender equality to achieve sustainable development by improving the well-being of society.

To this end, achieving gender equality in agriculture or significantly reducing its current magnitude through addressing gender gaps in access to modern production inputs (chemical fertilizer, improved seed, mechanization equipment), extension, and financial literacy, as well as improving the levels of human and social capacity building, could result in other non-negligible indirect gains in addition to gains in production, consumption, and poverty reduction. Achieving gender equality in agriculture carries wide-ranging indirect gains, encompassing economic growth, food security, climate resilience, social equity, and improved health and education outcomes. These benefits contribute to sustainable development and pave the way for inclusive and prosperous societies

## 5 Limitations of this paper and areas of further research

Despite the use of panel data for analysis and the two years (2017 and 2019) of national-level cross-sectional data, the study used household-level crop production and productivity data. The limitations of the household-level data compared to plot-level data might have disregarded women's contributions to farms in households headed by men. The influence of the structural or unexplained component of the agricultural productivity gap, which is far larger than the endowment component in this analysis, was not thoroughly investigated by the researcher. Therefore, the influence of unexplained or structural effects on productivity differences arising from only being female and assuming that they had equal access to endowment was not adequately considered by the researchers in this analysis. Consequently, future studies on the subject should concentrate more on the structural effects of gender differences between female-headed and male-headed households.

## CRediT authorship contribution statement

**Takele Abdisa:** Writing – review & editing, Writing – original draft, Visualization, Software, Project administration, Methodology, Investigation, Funding acquisition, Formal analysis, Data curation, Conceptualization. **Abule Mehare:** Writing – review & editing, Validation, Supervision, Software, Methodology, Formal analysis, Project administration. **Mekonnen B. Wakeyo:** Supervision, Methodology, Data curation, Conceptualization, Writing – review & editing, Writing – original draft, Visualization, Software, Formal analysis.

## Declaration of competing interest

The authors declare that they have no known competing financial interests or personal relationships that could have appeared to influence the work reported in this study.

## Data Availability

Data will be made available on request.

## Footnotes

1 One of the techniques most frequently employed in impact evaluation studies is Difference-in-Differences (DiD). The method, which combines before-after and treatment-control group comparisons, is intuitive and has been applied extensively in a variety of sectors, including public policy, management, health research, economics, and public health [31].
